# Thick artery–artery anastomoses delay the onset of selective fetal growth restriction in monochorionic diamniotic twins: a 7-year single-center cohort study

**DOI:** 10.3389/fmed.2023.1265875

**Published:** 2023-10-24

**Authors:** Xueju Wang, Luyao Li, Pengbo Yuan, Yangyu Zhao, Yuan Wei

**Affiliations:** Department of Obstetrics and Gynecology, Peking University Third Hospital, Beijing, China

**Keywords:** selective fetal growth restriction, onset time, placental territory, vascular anastomosis, monochorionic diamniotic twins

## Abstract

**Objective:**

This study aimed to investigate differences in placental characteristics between early- and late-onset selective fetal growth restriction (sFGR) in monochorionic diamniotic twins.

**Methods:**

A total of 253 patients with sFGR between April 2013 and April 2020 were retrospectively analyzed. Placental characteristics of early- and late-onset sFGR were compared.

**Results:**

The gestational age at diagnosis and delivery in the early-onset group was significantly less than that in the late-onset group [22.0 (16.9–23.9) and 28.4 (24.0, 36.3) weeks, *P* < 0.001; 33.1 ± 2.2 and 33.7 ± 2.5 weeks, *P* = 0.025]. The birth weight of normal growth and growth-restricted fetuses in the early-onset group was less than the late-onset group [1,990 ± 422 and 2,162 ± 525 g, *P* = 0.044; 1,320 ± 409 and 1,595 ± 519 g, *P* = 0.001]. The birthweight discordance ratio in the early-onset group was greater than the late-onset group (0.34 ± 0.12 and 0.29 ± 0.13, *P* = 0.001). The early-onset group had a significantly lower prevalence of sFGR type I than the late-onset group (37.5 and 62.0%, *P* = 0.018). The early-onset group had a significantly higher prevalence of sFGR type III than the late-onset group (30.4 and 12.7%, *P* = 0.048). The early-onset group had a lower prevalence of thick artery–artery anastomoses than the late-onset group (37.5 and 62.0%, *P* = 0.006). The placental territory discordance ratio in the early-onset group was higher than in the late-onset group [0.60 (0.01, 0.80) and 0.50 (0.01, 0.88), *P* = 0.018].

**Conclusion:**

Unequal placental territory is the cause for most of the late-onset sFGR. Thick artery–artery anastomoses may delay the onset time of these cases of sFGR.

## 1. Introduction

The incidence of selective fetal growth restriction (sFGR) in pregnancy with monochorionic diamniotic twins is ~10–15% (1). At present, an uneven distribution of placental territory and an aberrant insertion of the umbilical cord are deemed to be the causes of sFGR (1–3). Placental vascular anastomoses can affect the prognosis of the two fetuses to some extent (2, 4, 5). According to the onset time of sFGR, it can be categorized as early or late-onset sFGR. Previous studies showed that early-onset sFGR correlates with worse perinatal outcomes (6–8), and early-onset sFGR has a higher incidence of types II and III sFGR (7). As one of the complications resulting from the special placental structure in MCDA twins, the onset and prognosis of sFGR are closely related to its placenta. Lewi et al. found that more unequal placental territory is the cause of early-onset sFGR, whereas twin anemia polycythemia sequence (TAPS) between twins may be involved in some cases of late-onset sFGR (8). Our recent study also found that the sFGR placental anastomoses with TAPS were small and few, and sFGR with TAPS exhibited larger distances between umbilical cord insertions and little variation in placental share (9), which also supports the idea that TAPS is the reason for some cases of late-onset sFGR. However, the incidence of TAPS in MCDA twins is only 3–5% (10), and the cause of most cases of late-onset sFGR is still unclear. In addition, the cutoffs in previous studies were found to be different for early and late-onset sFGR (7, 8). So, to explore the variations between the two in terms of placental characteristics and to examine the effect of placental structure on the onset time of sFGR, we compared the early and late onset of sFGR for placental characteristics and pregnancy outcomes in this study.

## 2. Methods

This cohort study was carried out retrospectively from April 2013 to April 2020 on sFGR patients among MCDA twins who were admitted to the Obstetrics Department, Peking University Third Hospital. Excluded patients were those with twin anemia polycythemia sequence, twin-to-twin transfusion syndrome, one or two fetal deaths, fetal reductions, or suffering from a ruptured placenta post-delivery. The remaining patients were categorized into two groups according to the sFGR onset time. Following the study recently reported by Curado et al. (7), early- and late-onset sFGR were defined as diagnoses <24 and ≥24 weeks' gestation, respectively. The two groups were compared in terms of placental characteristics and pregnancy outcomes.

sFGR was diagnosed based on the ultrasound-estimated weight of either fetus being less than the 10th percentile of the corresponding gestational age (1, 7). The classification was as per an earlier reported criterion by Gratacos et al. (5). A normal spectrum of end-diastolic blood flow in the umbilical arteries of the fetus was defined as sFGR type I. A continuous absence or reversed end-diastolic velocity of the umbilical arteries of the fetus was defined as sFGR type II. Finally, an intermittently absent or reversed end-diastolic velocity of the umbilical arteries of the fetus was defined as sFGR type III (5). As the classifications of some cases in sFGR could change during pregnancy (11), we chose and recorded the final classification before delivery.

The diagnosis of TTTS was according to the combined presence of an MVP (maximum vertical pocket) of ≤2 cm in one sac and ≥8 cm in the other, irrespective of the gestational age during diagnosis (12). The diagnosis of antenatal TAPS was according to measurements of the middle cerebral artery peak systolic velocity (MCA-PSV) being >1.5 times the multiples of the median (MoM) in donors and <1.0 in recipients. The diagnosis of postnatal TAPS was based on large inter-twin differences in hemoglobin (>8 g/dL) and at least one of the following parameters, including minuscule placental anastomoses detected by the injection of the dye or >1.7 reticulocyte count ratio (13). The outcomes of pregnancy, including mode of delivery, birth weight, Apgar score after delivery, and pH of the umbilical artery, were noted. The neonatal asphyxia in China was diagnosed based on criteria of a 1 min Apgar score of <7 along with <7.2 pH of the umbilical artery (14). The calculation of the birthweight discordance ratio was as follows: the birthweight of the heavier fetus—birthweight of the lighter fetus/birthweight of the heavier fetus.

**Figure 1:**
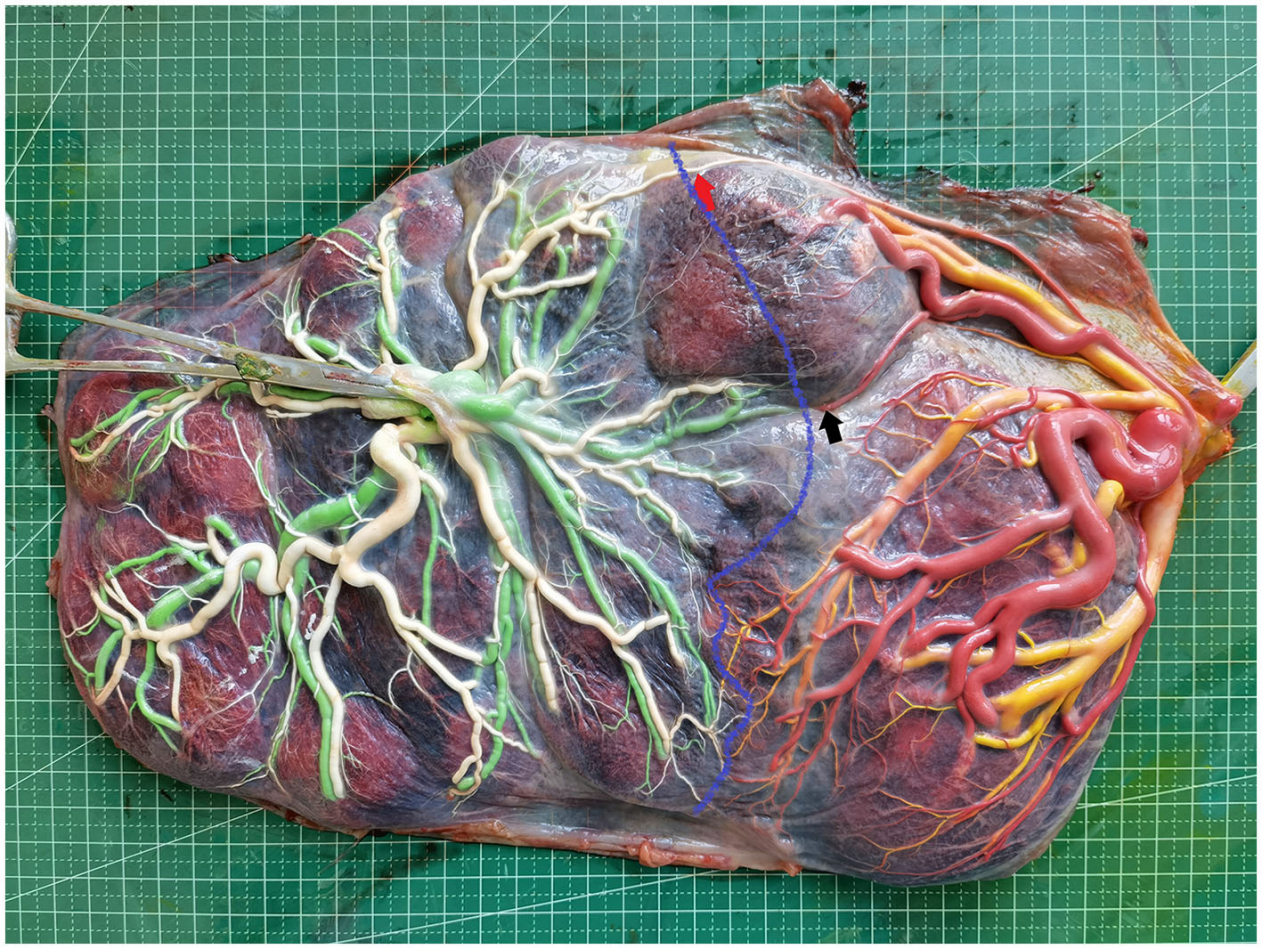
Image of placenta after dye injection (red arrow, AA anastomosis; black arrow, AV anastomosis; blue curve, vascular equator).

The placenta was stored in the refrigerator (at 4°C) and examined within 24 h. Placental dye injection was conducted as the protocol published by our center previously (15–18), injection was performed by 2 of the authors (XJ.W., LY.L.) blind of the diagnosis of placenta. After delivery, the amniotic membranes were removed, and each umbilical cord was cut 5 cm from its placental insertion site. The placental vessels were gently squeezed to clear out the blood clots. The umbilical vein and one of the umbilical arteries were then cannulated and clamped with an intravenous catheter. The placental vessels were injected with saline until all the branches were visible. The last step was performed using four 20-mL syringes, each filled with a distinctively colored dye (white, green, yellow, and red) to visualize the umbilical cord arteries and veins of the two fetuses. An image of the injected placentas captured using a high-resolution digital camera is shown in Figure 1.

**Figure 2:**
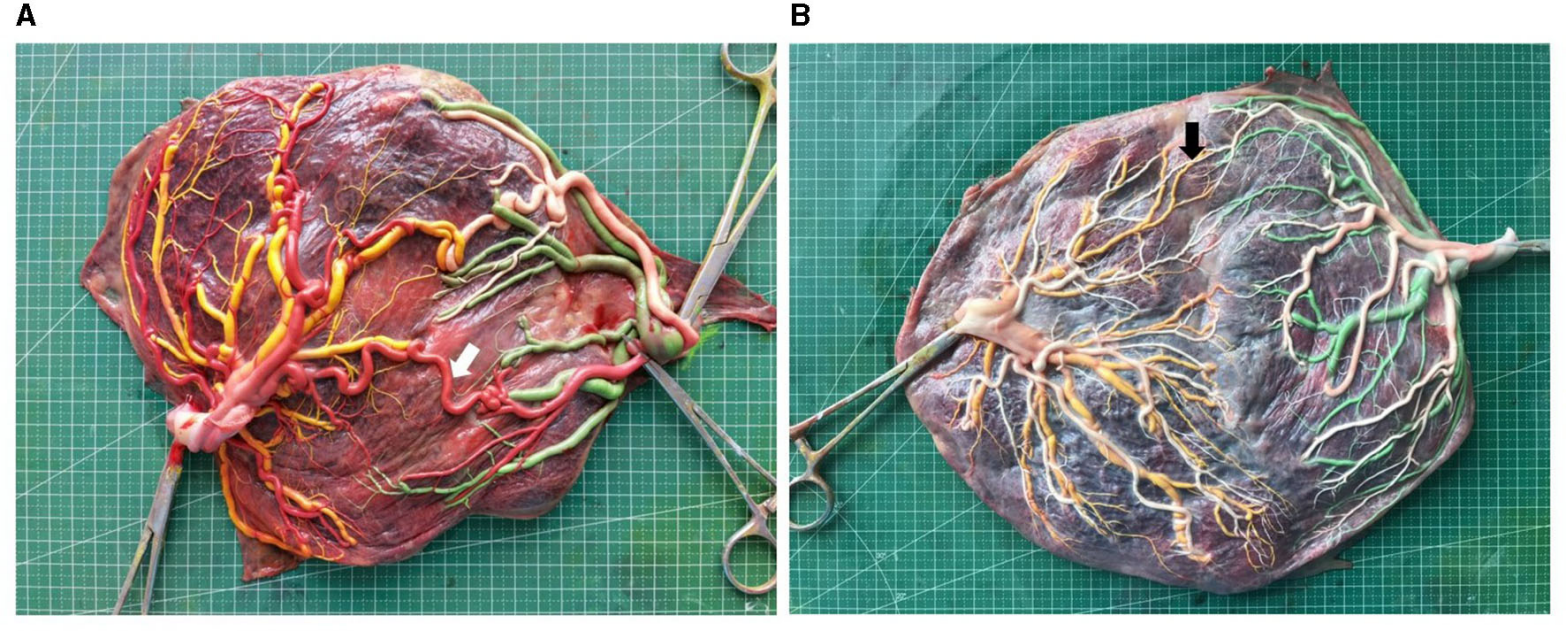
Image of placentas of early onset (placenta **A**) and late onset (placenta **B**) sFGR (white arrow, thick AA anastomosis; black arrow, small AA anastomosis. Placenta A sFGR type III diagnosed at 22^+0^ gestational weeks, delivered twins at 33^+2^ weeks with birthweight 2,050 and 1,500 g; placenta B sFGR type I diagnosed at 28^+0^ gestational weeks, delivered twins at 34^+5^ weeks with birthweight 2,220 and 1,850 g).

After saving, the analysis of placental images was performed using Image J V1.51j8 for Windows (NIH, Bethesda, MD, USA). Superficial vein-to-vein (VV) and artery-to-artery (AA) anastomoses were defined as direct external contact between two umbilical vessels that were homonymous. The caliber for both VV and AA anastomoses was determined as the least external diameter along the anastomosis course. Deep AV (artery-to-vein) anastomoses were identified where an artery that was not paired from one twin was found to have probed the chorionic plate near (<1.0 mm) the other twin's unpaired vein. The AV anastomosis diameter was designated as the diameter of the feeding artery at its narrow-most point. More than 2 mm in diameter of the vascular anastomosis is termed thick vascular anastomosis, as shown in Figure 2 (5, 18, 19). The longest distance between the placental parenchyma margins corresponded to the maximum placental diameter. The distance between the centers of insertion points of two umbilical cords was measured. However, the border of the adjacent placental parenchyma in the velamentous umbilical cords was chosen as the insertion point center. Marginal umbilical insertion involves the velamentous insertion and the distance of <1 cm between insertion points to the placental parenchyma margins. The umbilical insertion ratio was defined as the ratio of the distance between the centers of insertion points of two umbilical cords and the maximum placental diameter.

For each fetus, the placental territory was designated by the presence of specific dye margins. The expression of individual placental territories was performed as a percent of the total area and was calculated from acquired images using the Image J software. The vascular equator served as the placental territory boundary of the two fetuses and was defined based on the placement of all anastomoses. Then, after the delineation of each twin placental territory, the measurement was performed using freehand selection in the software (Figure 1).

The placental territory, vessel diameter, and distance between umbilical cords were assessed by a single observer (XJ.W). The intraobserver variation for the measurement was calculated in 10 randomly selected placentas. Each index was measured five times in each placenta. The coefficient of variation was calculated as the standard deviation of the observer's five estimations of each variable divided by the mean of the five estimations and multiplied by 100 (to give a percentage). The mean coefficient of variation of the measurements in the 10 placentas was used as a summary.

For statistical calculations, we used SPSS 24.0 software. The mean ± standard deviation or median (minimum and maximum) was used to present the results. To determine a normal distribution, the analysis of measurement data was carried out. In the case of a normal distribution of data in both groups, an independent-sample *t*-test was applied, followed by the presentation as the mean ± standard deviation. The Mann–Whitney (non-parametric) test was applied for other datasets and presented as the range and median. The chi-square test was used to check the enumeration data, whereas data from <40 cases were assessed using the Fisher's precision chi-square test. *P*-value in multiple comparisons was calculated using the Bonferroni correction; an adjusted *P*-value of <0.05 was considered to indicate statistical significance. Statistical significance was set at a *P*-value of <0.05.

## 3. Results

**Figure 3:**
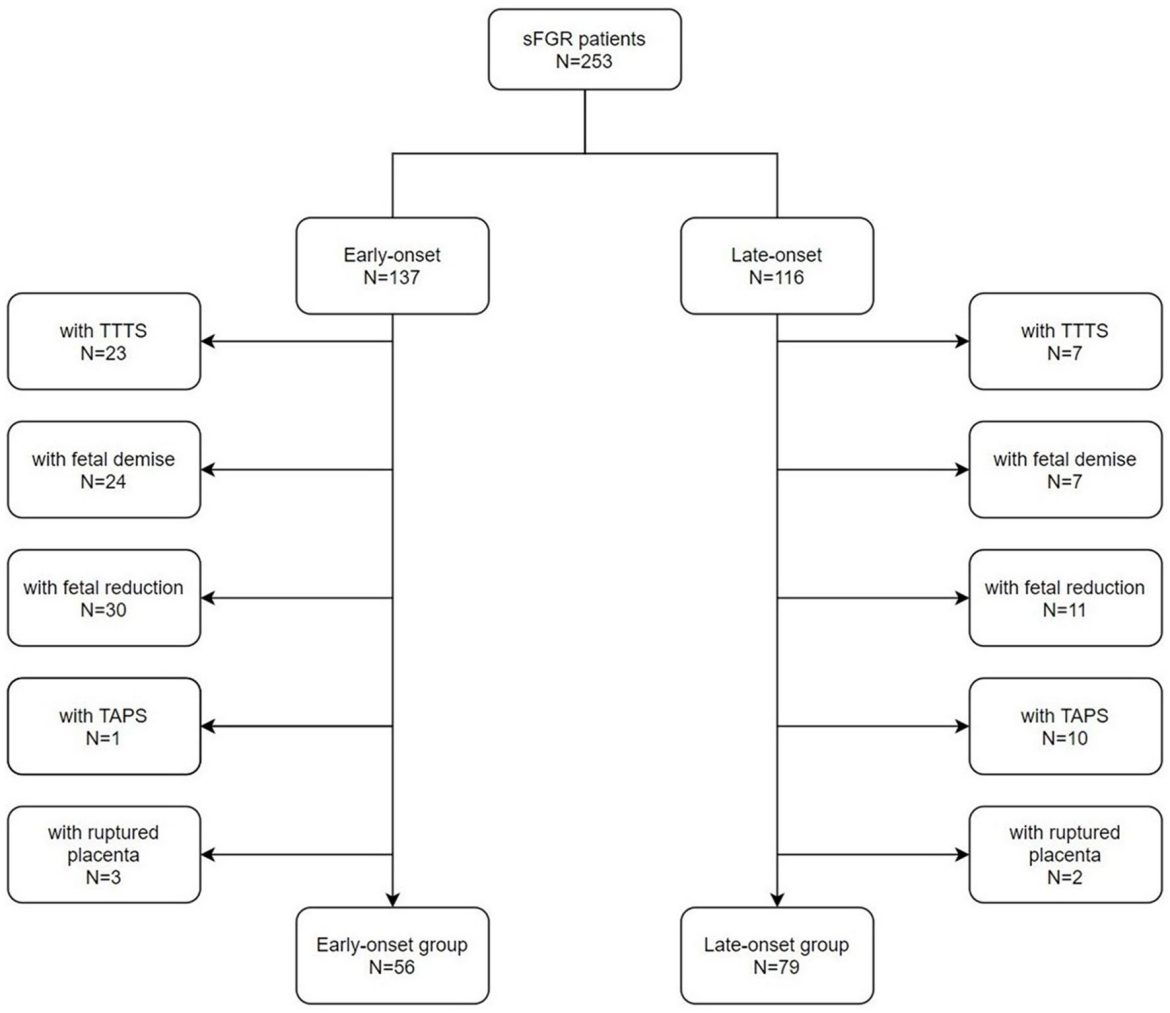
Flowchart of patients' inclusion.

A total of 253 patients diagnosed with MCDA twins (sFGR) were admitted from April 2013 to April 2020 to the Department of Obstetrics, Peking University Third Hospital, in this study. Figure 3 presents the flowchart of patients' inclusion.

Table 1 compares the pregnancy outcomes and general conditions of the two groups. The gestational age at diagnosis and delivery in the group with early onset was significantly less than the group with late onset. The birth weight of normal-growth fetuses and growth-restricted fetuses was both less than the group with late onset. The birthweight discordance ratio in the group with early onset was greater than in the group with late onset. The prevalence of sFGR type I in the group with early onset was significantly lower than in the group with late onset, and the group with early onset had a higher prevalence of sFGR type III than the group with late onset.

**Table 1:** Comparison of general characteristics and pregnancy outcomes of early- and late-onset sFGR.

	**Early-onset sFGR (*n* =56)**	**Late-onset sFGR (*n* =79)**	***P*-value**
Age (year)	30.3 ± 4.6	30.2 ± 5.0	0.974
Pregnancy-induced hypertension [cases (%)]	11 (19.6)	18 (22.8)	0.678
Gestational diabetes mellitus [cases (%)]	8 (14.3)	19 (24.1)	0.194
Gestational age at diagnosis (weeks)	22.0 (16.9, 23.9)	28.4 (24, 36.3)	< 0.001
**Final classification**			
sFGR type I [cases (%)]	21 (37.5)	49 (62.0)	0.009 0.018/0.999/0.048^*^
sFGR type II [cases (%)]	18 (32.1)	20 (25.3)	
sFGR type III [cases (%)]	17 (30.4)	10 (12.7)	
Gestational age at delivery (weeks)	33.1 ± 2.2	33.7 ± 2.5	0.025
**Delivery mode**			
Vaginal birth [cases (%)]	4	9	0.303
Cesarean section [cases (%)]	52	70	
Birthweight of normally growing fetuses (g)	1,990 ± 422	2,162 ± 525	0.044
Number of neonatal asphyxias-*n* (%)	7	5	0.235
Birthweight of growth-restricted fetuses (g)	1,320 ± 409	1,595 ± 519	0.001
Number of neonatal asphyxias-*n* (%)	7	4	0.109
Birthweight discordance ratio	0.34 ± 0.12	0.29 ± 0.13	0.001

^*^Adjusted *P*-value compared pairwise of sFGR types I, II, and III between the two groups.

The placental characteristics of the two groups were compared (Table 2). The intraobserver variation for the measurement of the placental territory, vessel diameter, and distance between umbilical cords was 1.3, 1.9, and 0.8%, respectively. The group with early onset had a lower prevalence of thick AA anastomoses than the group with late onset. Finally, the group with early onset had a higher placental territory discordance ratio than the group with late onset.

**Table 2:** Comparison between the two groups in terms of placental characteristics.

	**Early-onset sFGR (*n* =56)**	**Late-onset sFGR (*n* =79)**	***P*-value**
Prevalence of AA [cases (%)]	52 (92.9)	69 (87.3)	0.395
Prevalence of thick AA [cases (%)]	21 (37.5)	49 (62.0)	0.006
Total number of AA	1(1, 3)	1 (1, 2)	0.145
Total diameter of AA (mm)	3.2 ± 1.6	3.5 ± 1.9	0.725
Prevalence of AV [cases (%)]	51 (91.1)	76 (96.2)	0.276
Prevalence of thick AV [cases (%)]	15 (26.8)	26 (32.9)	0.569
Total number of AV	6 (1, 11)	3 (1, 9)	0.801
Total diameter of AV (mm)	5.2 ± 3.6	3.7 ± 3.2	0.320
Prevalence of VV [cases (%)]	14 (25.0)	12 (15.2)	0.186
Prevalence of thick VV [cases (%)]	10 (17.9)	11 (13.9)	0.632
Total number of VV	1 (1, 2)	1 (1, 2)	0.860
Total diameter of VV (mm)	2.9 ± 1.6	4.1 ± 2.3	0.164
Total number of anastomoses	9 (3, 15)	6 (3, 12)	0.710
Total diameter of anastomoses (mm)	11.2 ± 4.8	11.3 ± 5.9	0.557
Placental territory discordance ratio	0.60 (0.01, 0.80)	0.50 (0.01, 0.88)	0.018
Marginal umbilical insertion of normal growth fetuses [cases (%)]	3 (5.4)	10 (12.7)	0.237
Marginal umbilical insertion of restricted growth fetuses [cases (%)]	47 (83.9)	64 (81.0)	0.820
Umbilical insertion ratio	0.56 (0.10, 0.96)	0.62 (0.08, 0.98)	0.096

## 4. Discussion

This study compared the pregnancy outcomes of early and late-onset sFGR in MCDA twins and, for the first time, compared the characteristics of the placenta of early-onset and late-onset sFGR, excluding TAPS. As previous studies showed that TAPS is the reason for some cases of late-onset sFGR (8), and placental characteristics of sFGR complicated with TAPS are totally different from sFGR only cases (9). So, to rule out the influence of TAPS on the study, we excluded the cases of sFGR complicated with TAPS.

Our results showed that the group with early onset had a higher placental territory discordance ratio than the group with late onset, indicating the important role of unequal placental territory in the early-onset sFGR etiology. In agreement with the study by Lewi et al., they found the placentas of early-onset sFGR were more unequally shared (8). In combination with previous studies that compared the placental characteristics of sFGR and normal MCDA twins, it is well-known that unequal placental territory and marginal umbilical insertion are the two factors that cause sFGR in MCDA twins (1). However, we observed no significant difference between the two groups in the prevalence of marginal insertion of normal growth and restricted growth fetuses. So, based on our data and previous studies, we speculate that a more unequal placenta is the main cause of early-onset sFGR.

After determining the placental territory discordance, the median placental territory discordance ratio of late-onset sFGR was determined to be 0.5, which indicated that we cannot ignore the unequal placental territory in late-onset sFGR development. However, restricted growth appears later with such a huge placental territory discordance, and the factors influencing the onset time of such sFGR are still unclear. Placental territory and vascular anastomosis can influence the onset and development of sFGR (3, 5). Previous studies confirmed that thick AA anastomoses could balance the difference in hemodynamics between the two fetuses and play a protective role in the growth of restricted fetuses (2). Our study found that the prevalence of thick AA anastomoses in the group with early onset was lower than in the group with late onset, and there was no statistical difference in the total diameter of AA anastomoses in the two groups. However, few studies evaluate the differences in the diameter of anastomoses and the prevalence of thick AA anastomoses between early- and late-onset sFGR. Unlike the results of our study, Lewi et al. observed that the diameter of AA anastomoses in the early-onset sFGR was greater than that in the late-onset sFGR (3.66 ± 1.83 vs. 1.34 ± 1.15 mm, *p* < 0.01) (8), whereas the sample of their study was only 10 placentas in the two groups, respectively, and they did not exclude TAPS in the study, which could bring bias to the comparison of diameter of anastomoses because TAPS is characterized by remarkably few anastomoses of small size (<1 mm). So, we speculate that a higher prevalence of thick AA anastomoses in late-onset sFGR could balance the difference in hemodynamics between the two fetuses with placental territory discordance, which could delay the appearance of sFGR in such cases of MCDA twins. AA and VV anastomoses are bidirectional, also called superficial communications, which might be identified by color Doppler ultrasound (20). Fichera et al. reported that 63% of arterioarterial anastomoses were diagnosed using color Doppler imaging, mainly in anterior placentae (21). Spectral Doppler can be applied to identify the location of the vascular collision of AA anastomosis. In future, mapping of placental vascular anastomoses and placental territory discordance in monochorionic twins may help to identify the onset and prognosis of sFGR.

This study had some limitations; considering the requirements of placental dye injection, only those who received conservative treatment were included in the study. Placenta dye injection was conducted in sFGR with two live fetuses. Those with single fetal deaths and fetal reductions at the time of pregnancy were excluded, leading to a bias in the comparison.

## 5. Conclusion

Despite its retrospective nature and some limitations, this was a study with a large sample size for the comparison of placental characteristics of early-onset and late-onset sFGR. We speculate that unequal placental territory discordance is the cause of the majority of cases of late-onset sFGR in monochorionic diamniotic twins, whereas thick artery–artery anastomoses could be the factor delaying the onset time of these cases of sFGR.

## Data availability statement

The raw data supporting the conclusions of this article will be made available by the authors, without undue reservation.

## Ethics statement

The studies involving humans were approved by Ethics Committee of Peking University Third Hospital. The studies were conducted in accordance with the local legislation and institutional requirements. The participants provided their written informed consent to participate in this study.

## Author contributions

XW: Formal analysis, Investigation, Writing—original draft. LL: Conceptualization, Data curation, Formal analysis, Writing—review and editing. PY: Investigation, Methodology, Software, Writing—review and editing. YZ: Investigation, Supervision, Writing—review and editing. YW: Investigation, Supervision, Validation, Writing—review and editing.
